# Deep Soil Water-Use Determines the Yield Benefit of Long-Cycle Wheat

**DOI:** 10.3389/fpls.2020.00548

**Published:** 2020-05-15

**Authors:** Bonnie M. Flohr, James R. Hunt, John A. Kirkegaard, Brad Rheinheimer, Tony Swan, Laura Goward, John R. Evans, Melanie Bullock

**Affiliations:** ^1^The Commonwealth Scientific and Industrial Research Organisation (CSIRO) Agriculture and Food, Adelaide, SA, Australia; ^2^Department of Animal, Plant and Soil Sciences, La Trobe University, Melbourne, VIC, Australia; ^3^The Commonwealth Scientific and Industrial Research Organisation (CSIRO) Agriculture and Food, Canberra, ACT, Australia; ^4^Research School of Biology, The Australian National University, Canberra, ACT, Australia

**Keywords:** evaporation, fallow rainfall, harvest index, transpiration efficiency, water use efficiency

## Abstract

Wheat production in southern Australia is reliant on autumn (April-May) rainfall to germinate seeds and allow timely establishment. Reliance on autumn rainfall can be removed by sowing earlier than currently practiced and using late summer and early autumn rainfall to establish crops, but this requires slower developing cultivars to match life-cycle to seasonal conditions. While slow-developing wheat cultivars sown early in the sowing window (long-cycle), have in some cases increased yield in comparison to the more commonly grown fast-developing cultivars sown later (short-cycle), the yield response is variable between environments. In irrigated wheat in the sub-tropics, the variable response has been linked to ability to withstand water stress, but the mechanism behind this is unknown. We compared short- vs. long-cycle cultivars × time of sowing combinations over four seasons (2011, 2012, 2015, and 2016) at Temora, NSW, Australia. Two seasons (2011 and 2012) had above average summer fallow (December–March) rain, and two seasons had below average summer fallow rain (2015 and 2016). Initial plant available water in each season was 104, 91, 28, and 27 mm, respectively. Rainfall in the 30 days prior to flowering (approximating the critical period for yield determination) in each year was 8, 6, 14, and 190 mm, respectively. We only observed a yield benefit in long-cycle treatments in 2011 and 2012 seasons where there was (i) soil water stored at depth (ii) little rain during the critical period. The higher yield of long-cycle treatments could be attributed to greater deep soil water extraction (<1.0 m), dry-matter production and grain number. In 2015, there was little rain during the critical period, no water stored at depth and no difference between treatments. In 2016, high in-crop rainfall filled the soil profile, but high rainfall during the critical period removed crop reliance on deep water, and yields were equivalent. A simulation study extended our findings to demonstrate a median yield benefit in long-cycle treatments when the volume of starting soil water was increased. This work reveals environmental conditions that can be used to quantify the frequency of circumstances where long-cycle wheat will provide a yield advantage over current practice.

## Introduction

In southern Australia, fast developing spring wheat crops are traditionally established following the hot, dry summer fallow period on rains that fall in late autumn (April–May), and grow through the cool winter months to flower at an optimal time in early spring (September–October) during which collective damage from water stress, frost and heat are minimized (Richards, 1991; Zheng et al., 2012; Flohr et al., 2017). The drying trend recorded during austral autumn in semi-arid regions of the Southern Hemisphere (Pook et al., 2009; Cai and Cowan, 2013) unfortunately aligns with the optimal sowing window for short-cycle wheat cultivars, which combined with increasing farm size has prompted Australian growers to start sowing crops earlier (Fletcher et al., 2016; Flohr et al., 2018c).

Early sowing systems are facilitated by management that improves capture and storage of summer fallow rain including weed control, stubble retention and no-till farming to allow early germination, and the use of slow developing wheat cultivars (Hunt et al., 2013; Kirkegaard et al., 2014). Slow developing cultivars have a photoperiod or vernalization requirement that impedes progress through the crop’s life cycle such that flowering remains aligned to the optimal period despite earlier sowing dates (Pugsley, 1983). The combination of slow development and early sowing confers a longer crop life-cycle (long-cycle). Hunt et al. (2019) propose that by exploiting a much wider sowing window and longer growing season, long-cycle wheats can increase Australian national yields by 0.54 t/ha under current climate conditions, whilst managing the logistics of timely sowing on large farms. In response, and with the aid of a deeper understanding of genetic control, Australian breeders have begun to release a new generation of slow developing cultivars suitable for early sowing (Hunt, 2017).

Field experiments conducted over many decades have given conflicting results when comparing yield of long- and short-cycle treatments with synchronized flowering time across a range of environments (Penrose, 1993; Gomez-Macpherson and Richards, 1995; Penrose and Martin, 1997; Hunt, 2017; Peake et al., 2018). The study of Gomez-Macpherson and Richards (1995), and others reviewed by them (Batten and Khan, 1987; Connor et al., 1992) revealed that while long-cycle treatments accumulated more dry matter, yields were equivalent to short-cycle treatments due to lower harvest index (HI) in early sown cultivars. They postulated that while this could have been due to rapid growth in early sown crops depleting soil water so that little remained after flowering for grain fill (Passioura, 1977; Fischer, 1979), they ultimately suggested that long-cycle crops were taller and had more leaves. They proposed that competition for carbohydrates between the developing spike and elongating stem was responsible for reducing grain number and thus lower than expected yields and HI in early sown crops. In below average seasons in a high rainfall environment, Riffkin et al. (2003) reached a similar conclusion, finding over two seasons that long-cycle was inferior to short-cycle. Under high yielding irrigated conditions, Stapper and Fischer (1990) found yield of long-cycle was further reduced by a higher incidence of lodging. Also under irrigation and using isogenic lines, Fischer (2016) found that long-cycle treatments were equivalent or inferior to short-cycle treatments. The study of Coventry et al. (1993) demonstrated a yield advantage of early sown long-cycle treatments, but only in one (1986) of the two seasons (1985 and 1986) studied.

Peake et al. (2018) suggested that in most of these studies that a deficiency of nitrogen may have limited grain yield, therefore long-cycle wheats with greater dry matter may have been discriminated against by experiencing greater nitrogen stress. Peake et al. (2018) applied best practice agronomy under sub-tropical irrigated conditions and achieved synchronized flowering between short and long-cycle treatments in multiple site and year experiments between 2014 and 2016 (7 experiments in total). Results from these experiments showed a yield advantage (0.5–1.5 t ha^–1^) in 70% of experiments for long-cycle treatments over short-cycle treatments. Simulation modeling was used to infer that long-cycle had a yield advantage when imperfect irrigation timing resulted in crops experiencing short periods of drought stress. They concluded that the yield advantage was due to long-cycle treatments being better able to withstand drought stress, but did not take the measurements necessary to identify the mechanisms responsible.

Hunt (2017) hypothesized that long-cycle treatments may only have a yield advantage in seasons when the soil profile has water that is accessible at depth, and limited water at shallow depths due to limited rainfall so that the potentially deeper root growth of long cycle treatments becomes advantageous. Whilst the study of Peake et al. (2018) supports this hypothesis, it has not been tested in experiments with measurements of soil water and dry matter accumulation. The aim of this study was to clarify the environmental conditions that confer an advantage to long-cycle crops in a rain-fed environment, and identify the mechanisms responsible for any observed yield advantages.

## Method

### Field Sites

Field experiments were conducted in four seasons (2011, 2012, 2015, and 2016) at sites near Temora, New South Wales (NSW, Table 1). Temora has a mean annual rainfall of 520 mm (1963–2013) with 312 mm on average falling during the wheat growing season (April–October) and 208 mm falling during the summer fallow period (November–March). Experiments were split-plot (whole plot = time of sowing, sub-plot = cultivar) with four replicates, and either randomized complete block or row: column designs. Sowing date was randomized within replicates. Soil type at all sites the was a red chromosol (Isbell, 2002), with plant available water capacity (PAWC) as per Table 1. In 2015 and 2016 the soil type was per profile number Temora No. 913 in the APSoil database^1^. Rainfall summaries and site details for the four experimental growing seasons are reported in Table 1.

**TABLE 1:** Site details for each year of the experiment, including soil plant available water capacity (PAWC), plant available water (PAW) at sowing, summer fallow (Dec-Mar), growing season (Apr-Oct) rainfall and rainfall in the 30 days prior to the start of the optimal flowering period (OFP).

**Site detail**	**Year**			
	**2011**	**2012**	**2015**	**2016**
Location	S 34.49°, E 147.51°	S 34.73°, E 147.54°	S 34.41°, E 147.53°	S 34.41°, E 147.53°
PAWC (mm) and depth of measurement	203 (1.85 m, Hunt et al., 2016)	196 (1.85 m)	206 (1.6 m, Wang et al., 2018)	206 (1.6 m, Wang et al., 2018)
PAW at sowing (mm)	104	91	28	27
Summer fallow (Dec-Mar) rainfall (mm)	408	386	206	113
Growing season (Apr-Oct) rainfall (mm)	200	169	279	588
Rainfall 30 days prior start of OFP (mm)	8	6	14	190
Soil mineral nitrogen at sowing (kg/ha)	207	52	209	89
N fertilizer (kg/ha N) applied as urea	74	171	46	198

### Cultivar × Sowing Date Selections

Each experiment had four cultivars that were selected as being highest yielding milling (Australian Hard) spring wheat cultivars for four development types (Table 2, fast, mid, slow, and very slow developing) based on yield performance in National Variety Trials for south eastern NSW (ACAS, 2007). The alleles of major development genes for each cultivar are as described by Bloomfield et al. (2018) and are presented in Table 2. Alleles for the major genes indicate that the four cultivar’s development is moderated from fastest to slowest by an increasing number of vernalization and photoperiod sensitive alleles. In 2015 and 2016 a fast spring (Sunstate) and very slow spring (W16A) near-isogenic pair (Hunt et al., 2019) were also included in experiments.

**TABLE 2:** Genotypes used in experiments and alleles at five major development loci using nomenclature after Bloomfield et al. (2018).

**Cycle length**	**Development**	**Cultivar**	**Ppd-B1**	**Ppd-D1**	**Vrn-A1**	**Vrn-B1**	**Vrn-D1**
Short	Fast	Lincoln	b	a	a	a	v
	Fast	Condo	a	a	v	a	a
	Fast	Sunstate	a	a	v	a	a
	Mid	Gregory	b	a	v	v	a
Long	Slow	Bolac	b	a	a	v	v
	Very slow	Eaglehawk	b	b	b	v	a
	Very slow	W16A	b	b	e	a	a

**For Ppd genes “a” indicates insensitive allele and “b” indicates sensitive. For Vrn genes “a” and “b” denote different spring alleles, and “v” winter (wild type) allele.**

### Crop Management

All crops were direct-drilled in small plots (1.8 m × 9 m) on 305 mm row spacing with press wheels to give six crop rows per plot. Seeding depth was ∼40 mm depending on seed bed moisture. Each experiment had 4 sowing dates spaced at 10 day intervals commencing in mid-April and ending in mid-May, but only data for the treatments that flowered concurrently and within the optimal flowering period for Temora (25 September–10 October as per Flohr et al., 2017) are shown (Table 3). Sowing date is defined as the calendar date at which seeds become imbibed and begin the process of germination, i.e., either the date on which they are planted into a moist seed bed, or the date on which they received rainfall/irrigation after being sown into a dry seed bed. Seed bed moisture was insufficient to establish all sowing dates at Temora in 2011 and 2016, and at the early and mid-May sowings either 8 mm (2011) or 15 mm (2016) of water was applied to the sown furrow using drip irrigation. As this small amount was applied to very dry soil and did not penetrate deeply, it is assumed not to have contributed to crop transpiration and yield. In all experiments, chemical fertilizers and pesticides were applied such that nutrient limitations, weeds, pests or diseases did not limit yield. Grain protein in all experiments exceeded 11.1% indicating N deficiency was unlikely (Goos et al., 1982; Holford et al., 1992).

**TABLE 3:** Target and actual sowing dates in 2011, 2012, 2015, and 2016.

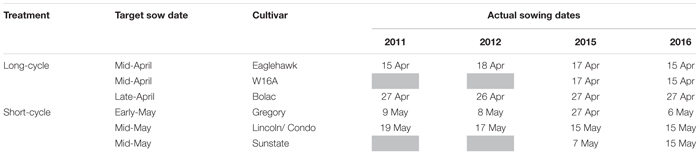

To compare crop development rates between the four experimental seasons, degree days, i.e., Thermal time (TT) = Σ ((T_*min*_ + T_*max*_)/2) − T_*base*_ accumulated using 0°C as the base temperature and starting from the first sowing date were calculated for each season, using data available from the Commonwealth Bureau of Meteorology (BoM) website (Australian Government, 2017).

### Management of Treatments to Manipulate Harvest Index

Previous experiments in southern Australia have demonstrated that long-cycle wheat has lower HI than short-cycle treatments (Batten and Khan, 1987; Connor et al., 1992; Gomez-Macpherson and Richards, 1995; Riffkin et al., 2003). Modified agronomic management can be used to alter carbohydrate partitioning and early dry matter accumulation in order to improve HI. To this end, two plant density treatments (“high” = ∼100 plants/m^2^ and “low” = ∼50 plants/m^2^) were imposed at the optimal sowing date for each cultivar in 2011 and 2012 experiments. In 2011 a plant growth regulator (PGR) treatment designed to reduce stem height and consisting of 50 g/ha trinexapac-ethyl + 757 g/ha chlormequat chloride applied at development stage 30 (Tottman, 1987) was compared to a control in a factorial design with plant density at the optimal time of sowing of each cultivar. In 2012, two defoliation treatments (control and Z30 defoliation as per Kirkegaard et al., 2015) were used in a factorial design with plant density at the optimal time of sowing of each cultivar. The effect of all treatments on yield and other parameters were uniformly small and often not significant. The effect of the treatments on all data were analyzed using either one or two-way analysis of variance (ANOVA) assuming a split plot design in the GenStat 15 software package (VSN International, 2013). Significance is assumed at the 95% confidence level. If management treatments to increase HI had a significant interaction with cultivar x sowing date combinations on any variate, they were not included in the analysis of that variate and only control treatments (“high” plant density, no PGRs and no defoliation) were used. If the interaction was not significant, then pooled means incorporating the management treatments were used in the comparisons and figures below.

### Plant and Soil Measurements

Day of flowering was recorded as the date when 50% of the spikes in each plot had at least one visible anther DC65 (Z65, Zadoks et al., 1974). Dry matter (DM) was measured at Z89 (maturity) by cutting all above ground plant parts in a quadrat 0.39 × 1.2 m (four middle rows from plots) in 2011, 2012, and 2016, and a quadrat 0.83 × 1.2 m (four middle rows from plots) in 2016 per replicate. At DC69 and DC89, 20 stems were partitioned into stem, leaf, spike and dried at 70°C for at least 48 h to record a dry weight. Stem weight was not recorded in 2012. Individual grain weight was measured by weighing 200 grains dried at 70 °C for at least 48 h. Harvest index was calculated as the ratio of the grain to the total DM of the sample taken at maturity. In 2012 grain yields were measured by machine harvest of the inside four rows of six row plots and are reported at oven-dry moisture content. In 2011, 2015, and 2016 yield was measured by hand harvesting and threshing a quadrat of either 0.39 × 1.2 m (2011 and 2015) or 0.83 × 1.2 m (2016) taken from the inside four rows of six row plots and are also reported at oven-dry moisture content. Harvest grain moisture was determined via Near Infrared (NIR) technology, and grain yield was divided by grain weight (also 0% moisture) to calculate grain/m^2^.

A neutron moisture meter (NMM) access tube was installed centrally to a depth of 1.8 m in April of each year to allow in-crop measurements of soil water. Recordings using an NMM (CPN International, Martinez, CA) were taken at 0.1 m increments to depth of 1.6 m and volumetric water content was calculated using an existing calibration (Hunt et al., 2016). To determine plant available water (PAW) in April, the change in soil water content between sowing and crop maturity was used to estimate the crop lower limit.

In 2011 and 2012 groundcover was estimated using regular readings of NDVI recorded using a GreenSeeker^®^ (Trimble Inc., Sunnyvale CA), and then used to estimate PAR light interception based on an existing unpublished relationship developed for wheat at Temora (PAR = 1.60^∗^NDVI - 0.39, *R*^2^ = 0.92). In 2015 and 2016 canopy light interception PAR was recorded around solar noon using a ceptometer (AccuPAR LP-80; Pullman, WA, United States) at 4 positions per plot at the time of DC39 and DC70 DM sampling. Values of daily fractional PAR interception were obtained by interpolation between readings of interception (Monteith, 1972) and then used to estimate daily soil evaporation (E_*s*_) based on FAO56 values of potential evapotranspiration (obtained from www.longpaddock.qld.gov.au, ET_*o*_) and days since last rain fall (d) after the method used by Siddique et al. (1990) where

E=sET(1/d)o*

Daily evaporation under each cultivar (E_*sc*_) was then estimated from

E=s⁢cE(1-a)s*

where *a* is the daily interpolated PAR interception.

Daily estimates of E_*s*_ were summed from the day on which initial NMM soil water measurements were made (early April) to maturity to estimate seasonal soil evaporation. Seasonal transpiration was calculated as the difference between seasonal crop water use and evapotranspiration (i.e., crop water use - E_*s*__*c*_), and this was used to calculate transpiration efficiency (TE) for dry matter. The NMM measurement was taken on the same date regardless of sowing date. Water use efficiency (WUE) was calculated by dividing total dry matter at maturity by total crop water use. NMM measurements made at flowering were used to calculate the proportion of water used pre- and post-flowering. In 2015 and 2016 these were calculated for the near-isogenic lines only (W16A and Sunstate), as neutron moisture meter tubes were only installed in these treatments. Evapotranspiration is defined as rainfall plus the change in soil water content between sowing and crop maturity.

### Statistical Analysis

Similar to the analysis of Peake et al. (2018), treatments were grouped by long- and short-cycle cultivars that flowered synchronously during the optimal flowering period defined by Flohr et al. (2017). In each year, a two-sample *t*-test (GenStat 19 user interface, VSN International, 2013) was used to test for significant differences between pooled means of long- and short-cycle treatments. For each season, long-cycle and short-cycle treatment yields were used to calculate the yield difference of long-cycle treatments over short-cycle treatments, which were plotted against the soil water extraction from between 1.0 and 1.6 m depth for each block. Linear regression analysis was used to determine relationships between the yield benefit of long-cycle treatments and soil water extraction below 1.0 m, and between HI and proportion of water used after flowering. Non-linear regression analysis was used to fit grain yield and grain number data.

### Investigation of Yield Differences Between Long and Short Cycle Treatments Under Different Levels of Starting Soil Water Using Simulation

The Agricultural Production Systems Simulator (Holzworth et al., 2014) was used to evaluate yield differences between long- and short-cycle treatments under different levels of starting soil water in seasons between 1997 and 2016. The APSIM modules used in the analysis were Wheat (wheat crop growth and development), SoilWat (soil water balance), SoilN (soil nitrogen dynamics), SurfaceOM (surface residue dynamics), and Manager (management rules) as described by Hunt and Kirkegaard (2011). In order to validate APSIM’s ability to simulate the differences between long- and short-cycle treatments, it was parameterized for the Eaglehawk sown mid-April (long-cycle) and Lincoln sown mid-May (short-cycle) treatments in the field experiments described above. Soil input data for the 2011 site were derived from a detailed characterization conducted at the experimental site and is reported by Hunt et al. (2016). Soil data for 2012 were derived from measurements of drained upper limit and crop lower limit made in an adjacent field reported by Kirkegaard et al. (1994). Initial soil water and mineral N were measured from intact soil cores taken across each site to a depth of 1.65 m and segmented 0–5 cm then every 10 cm below that. Crop specific inputs (sowing depth, plant density, row spacing) were used from measurements recorded in the experiment. The parameterization for Eaglehawk available in the released version of APSIM gave good agreement between observed and simulated flowering dates and was used to simulate that cultivar. The released parameterization for Lincoln was found to under predict flowering time by ∼10 days, and a new parameterization was created to optimize flowering date with observations (Supplementary Figure S1). Daily minimum and maximum temperatures were recorded using iButton datalogers (Maxim Integrated, San Jose, CA) housed in a wooden radiation screen at 1.5 m. Daily rainfall was recorded using a tipping bucket rain gage. All other meteorological inputs were obtained from the SILO Patched Point Dataset (Jeffrey et al., 2001) for the nearby Australian Bureau of Meteorology station 073038 (Temora Research Station). The APSIM yield prediction assumes phosphorus and all nutrients other than N are non-limiting and does not incorporate the effects of the presence of pests, disease, weeds, or heat and frost shock.

To extend the field experiments described here across a greater range of seasons and levels of starting soil water, a simulation experiment was conducted with short- (Lincoln sown 15 May) and long-cycle (Eaglehawk sown 15 April) treatments at two locations (Temora and Griffith) defined by SILO Patched Point Dataset for Australian Bureau of Meteorology stations 073038 (Temora Research Station) and 075041 (Griffith Airport AWS). Griffith was selected as the second site as it has a similar soil type and latitude as Temora but is warmer (mean annual temperature 17.0 vs. 15.5°C) and drier, receiving an average (1958–2018) annual rainfall of 401 mm, with 237 mm falling during the growing season. The Temora 2011 site soil type was used at both sites. Crop growth and yield was simulated at different levels of starting soil water by resetting soil water at 5, 25, 50, 75, and 100% of plant available water capacity (203 mm) filled from the top each year of the simulation. There was also a sixth “natural” treatment in which the soil water was set at 0% of PAWC in the first year of the simulation and not reset after that. The years 1987–2016 were simulated, but the first 10 years discarded in order to allow soil water in the “natural” treatment to equilibrate. In all simulations NO_3_ in the top three soil layers were maintained above 50 kg/ha such that N supply did not limit yield. Crops in both treatments were planted at 100 plants m^–^^2^ on 305 mm row spacing. Output variables reported included yield, root_depth, seasonal soil evaporation, seasonal transpiration.

## Results

### Growing Conditions

The four growing seasons studied contrasted in their pattern of seasonal water availability (Table 1). NMM measurements made on 7, 3, 14, and 15 April in each growing season, respectively, determined average initial PAW across the site to be 104, 91, 28, and 27 mm. Rainfall 30 days prior to the start of the optimal flowering period (25 September as per Flohr et al., 2017) in each year is reported in Table 1. This period is assumed to correspond to the critical period for yield determination in wheat (Fischer, 1985). The disparate patterns of rainfall gave three different season types;

1. Deep stored soil water present (from summer rain) and drought during critical period (2011 & 2012).
2. No deep stored soil water and drought during the critical period (2015).
3. Deep stored soil water present (from in-crop rain) and no drought during the critical period (2016).

**FIGURE 1:**
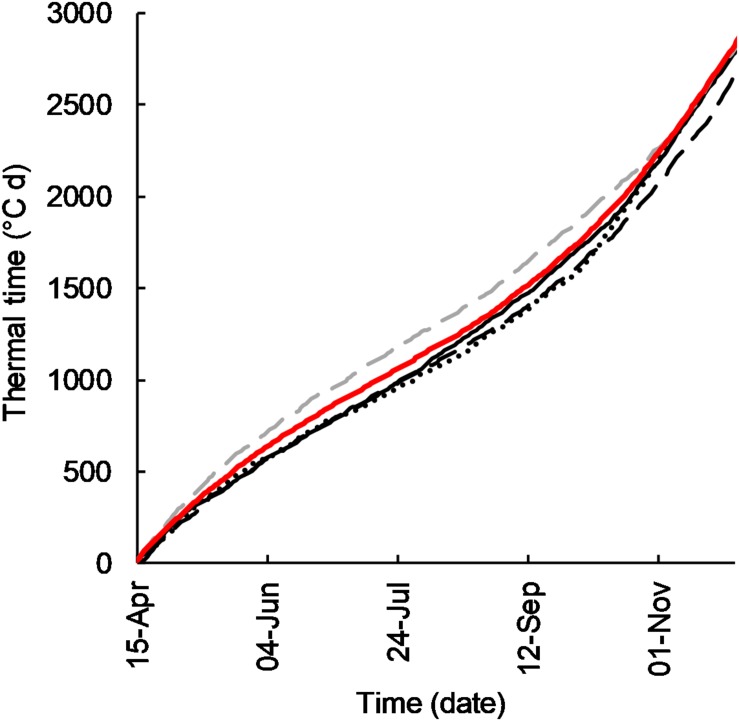
Cumulative thermal time (°C d) for Temora, NSW in growing seasons 2011 (-), 2012 (- -), 2015 (∙∙∙), 2016 (- -), and average 2005–2016 (-).

The monthly TT accumulation (°C) in the year of experiment for each site is summarized in Figure 1. Accumulation of thermal time was near average in 2011, lower than average in 2012 and 2015, and above average in 2016.

### Synchronization of Flowering Dates and Grain Yield

**FIGURE 2:**
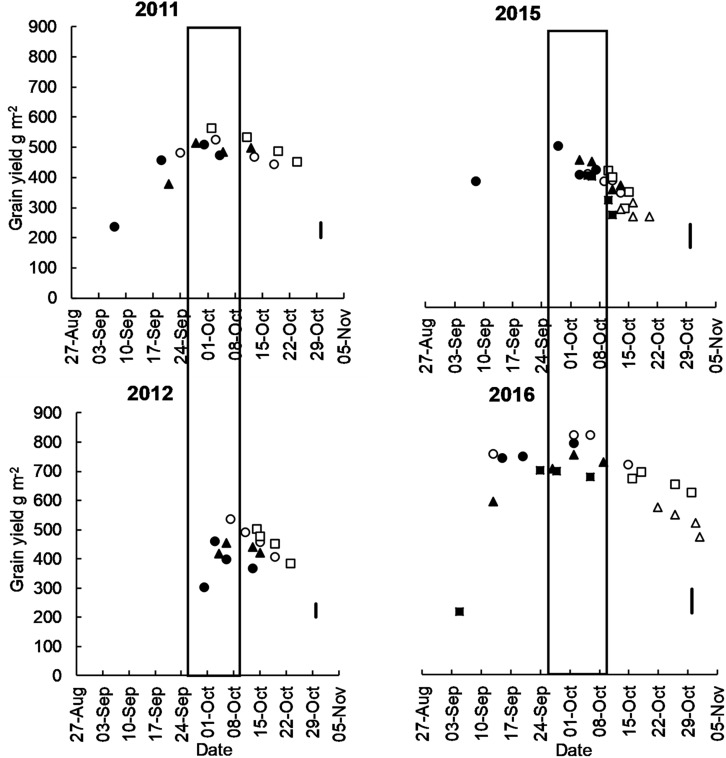
Mean grain yield of long cycle (Eaglehawk □, W16A △, Bolac ○) and short cycle (Gregory ▲, Lincoln/Condo ⚫, Sunstate ■) cultivars plotted against flowering date at Temora in 2011, 2012, 2015, and 2016. Box represents the optimal flowering period as per Flohr et al. (2017). Each marker represents a different time of sowing (from mid-April to mid-May) and sowing date moves later from left to right. The vertical line is LSD, and the *P*-Value of ToS x cultivar interactions at all sites is <0.001.

Whilst the precise target sowing dates were not achieved (Table 3), flowering of cultivars sown at their optimal time was satisfactorily concurrent in 2011, 2012, and 2015 (Table 4). In 2011 and 2012 flowering occurred within a range of 3 days, and in 2015 within 8 days. In 2016 flowering dates ranged by 20 days. In 2016 the very slow developing types (EGA Eaglehawk and W16A) flowered 6–12 days after the optimal flowering period when sown at the earliest sowing date, all other development types flowering within the optimal period (25 September–10 October as per Flohr et al., 2017). This behavior was possibly due to the warmer than average conditions experienced that season (Figure 1) hastening development of most cultivars, whilst the strong photoperiod sensitivity of EGA Eaglehawk and W16A impeded development despite above average temperatures. As W16A and EGA Eaglehawk did not flower at a comparable time to the other cultivars nor within the optimal period in 2016 (Figure 2), they were both excluded from the long vs. short cycle analysis for all variates except crop water-use in that year. The short cycle NIL pair of W16A (Sunstate) was also excluded so as not to bias in favor of long-cycle by retaining a line that is not an elite cultivar. W16A and Sunstate were still used to calculate crop water use in 2016 as these were the only treatments in which NMM tubes were installed.

**TABLE 4:** Flowering dates (Z65 – 50% of ears with anthers extruded) for the different maturity groups in 2011, 2012, 2015, and 2016.

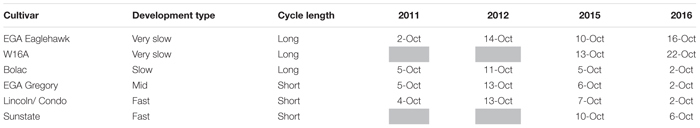

In all growing seasons, highest yields were achieved in treatments that flowered within the optimal flowering period defined by Flohr et al. (2017) (Figure 2).

### Grain Yield, Number, Grain Size, Dry Matter, and HI

In 2011 and 2012 long-cycle treatments yielded significantly more than short-cycle treatments. In 2015 and 2016 there was no significant difference in grain yield between short- and long-cycle treatments (Table 5). In all growing seasons long-cycle treatments had higher grain number and this was significant in three of four seasons. In 2011 and 2012 growing seasons, long-cycle treatments accumulated more dry-matter than short-cycle treatments. Higher yields in long-cycle treatments were due to higher grain number at similar (2012) or even lower grain weight (2011, Table 5). Higher grain number in long-cycle cultivars in 2016 did not result in higher grain yield, and short-cycle treatments had greater grain weight (Table 5). There was no significant difference in HI or stem weight % of total dry-matter at flowering in all growing seasons.

**TABLE 5:** Mean values for long- and short-cycle treatments grown in Temora, for dry-matter at maturity, grain yield, harvest index, grain number, stem weight at flowering and grain weight in each growing season.

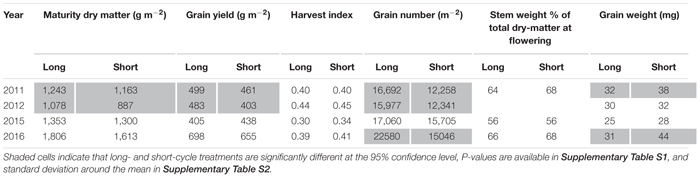

**FIGURE 3:**
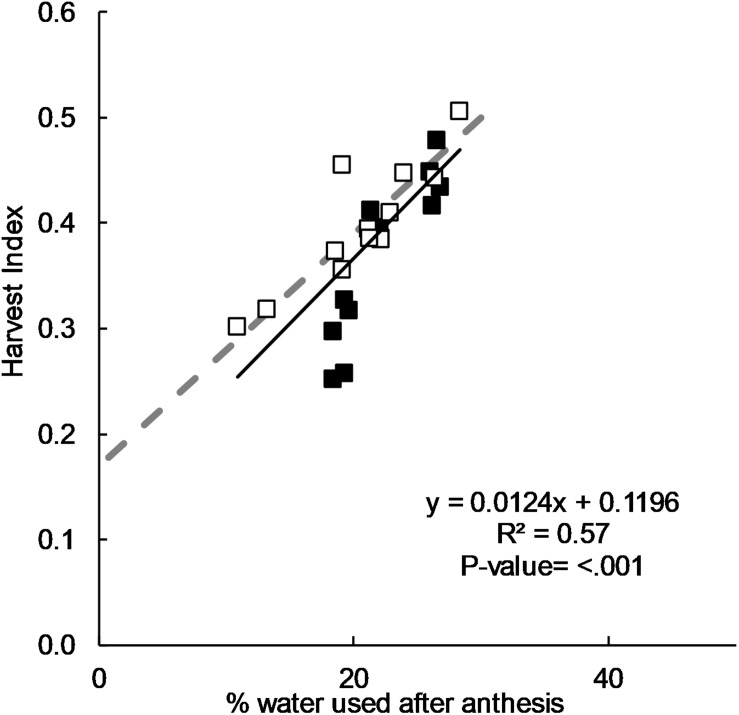
Relationship between harvest index and post-flowering water use as a ratio of seasonal water use for long-cycle (■) and short-cycle (□) cultivars grown in 2011, 2012, 2015, and 2016. The same relationship postulated by Passioura (1977)(- -).

There was a positive correlation between HI and the percentage of water used after flowering (Figure 3) and this was in good agreement with the relationship derived by Passioura (1977) from pot experiments. A HI of 0.50 was observed when 35% of water was used after flowering, and a progressively lower HI as the percentage of water used after flowering decreased.

### Crop Water Use

In the presence of deep stored soil water and terminal drought, there were no significant difference in crop water use in 2011, but in 2012 long-cycle treatments used more water than short-cycle (Table 6). This was due to deeper root growth (see Kirkegaard et al., 2015 for root measurements from these experiments in 2012) in the long-cycle treatments. Long-cycle treatments lost less water to evaporation in all (not significant in 2016) seasons due to earlier canopy development (Table 6). WUE for dry matter was higher for the long-cycle treatments compared to short-cycle treatments in 2011 and 2012, due to both less evaporation and higher TE for dry matter in 2012.

**TABLE 6:** Mean values for long- and short-cycle treatments grown in Temora for crop water use, estimated evaporation, post-flowering water use, transpiration efficiency (TE) for dry matter at maturity and water use efficiency (WUE) for grain yield in each growing season.

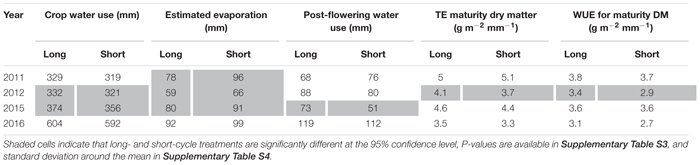

In a growing season with no deep stored soil water and drought during the critical period (2015), and a season with above average rainfall (2016), there was also greater crop water use by long-cycle treatments (not significant in 2016), but this did not result in a higher grain yield (Table 6).

### Use of Stored Soil Water and Yield Response

**FIGURE 4:**
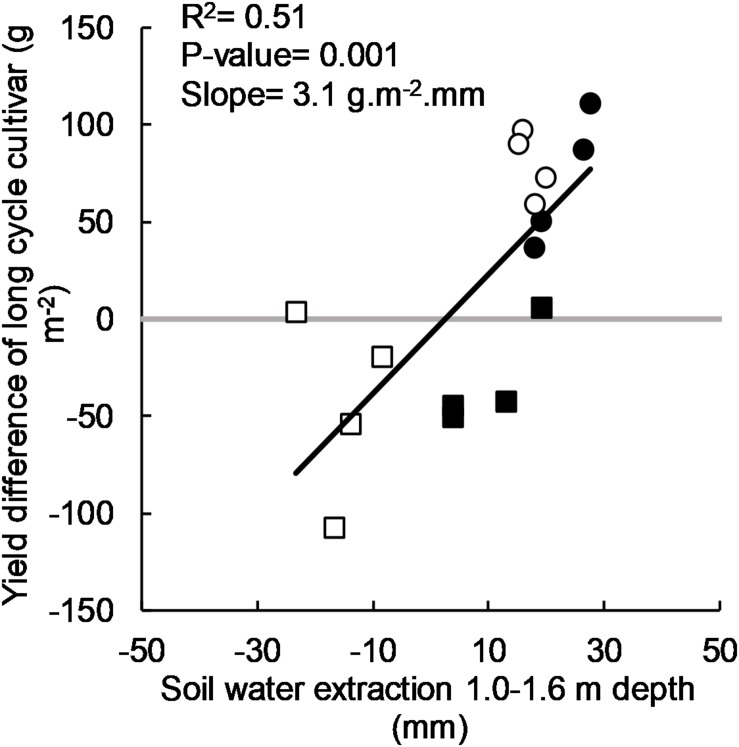
Yield difference of long-cycle cultivars (Eaglehawk, Bolac, W16A) over short-cycle cultivars (Gregory, Lincoln, Condo, Sunstate) in 2011 (•), 2012 (○), 2015 (•), and 2016 (□) and soil water extracted from 1.0 to 1.6 m depth by the long-cycle treatment. Each symbol represents values for individual blocks (replicates) within each experiment. *P*-value and *R*^2^ relates to the regression fitted to the data.

There was a significant positive linear relationship between the yield benefit of long-cycle treatments and soil water used below 1.0 m (Figure 4). Although there was variance unaccounted for (*R*^2^ = 0.51), yield of long-cycle cultivars increased by 3.1 g m^–2^mm^–1^ (or 31 kg ha^–1^mm^–1^) of soil water extracted below 1.0 m.

### Grain Number Drives Increased Grain Yield in Long-Cycle Treatments

**FIGURE 5:**
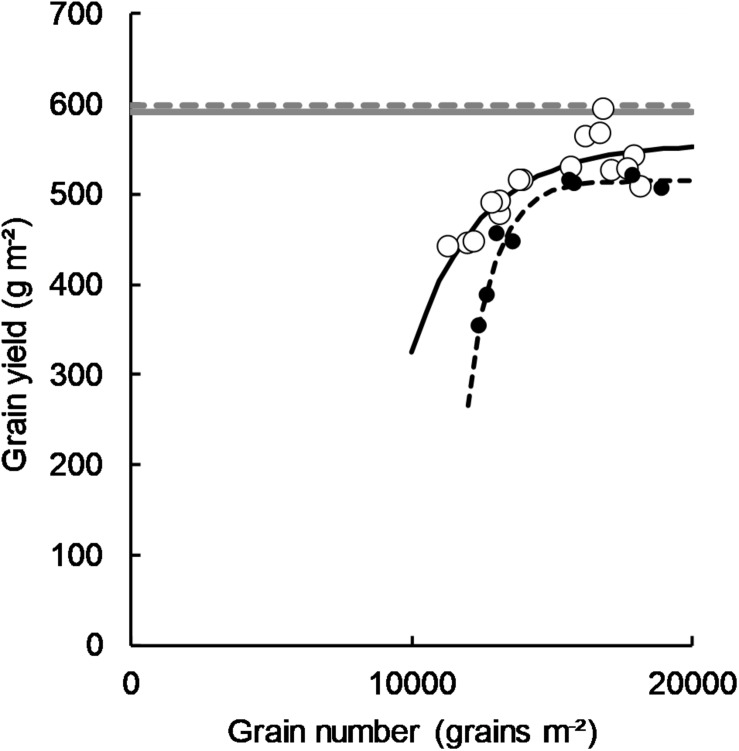
Grain number plotted against grain yield in 2011 (○, *y* = 556-14084*(0.999^*X*^), *P* = <0.001, *R*^2^ = 0.72) and 2012 (■, *y* = 515-5.3 × 10^7^*(0.999^*X*^), *P* = <0.001, *R*^2^ = 0.94) referenced against the water limited yield potential as per Sadras and Angus (2006) for each 2011 (solid gray line) and 2012 (broken gray line). Data points are contributed by four sowing time x cultivar treatments (two long-cycle, two short-cycle) and the management applied to increase HI as per section Management of Treatments to Manipulate Harvest Index (plant density and PGR treatments in 2011 and density treatment in 2012).

In both 2011 and 2012, there was an asymptotic relationship between grain number and grain yield (Figure 5). In both years, the asymptotes approached the water limited potential yield calculated for each site as per Sadras and Angus (2006) where water limited yield potential (PY_w_) = (ET-60)^∗^22.

### Simulation of Long and Short Cycle Treatments Under Varying Levels of Starting Water

**FIGURE 6:**
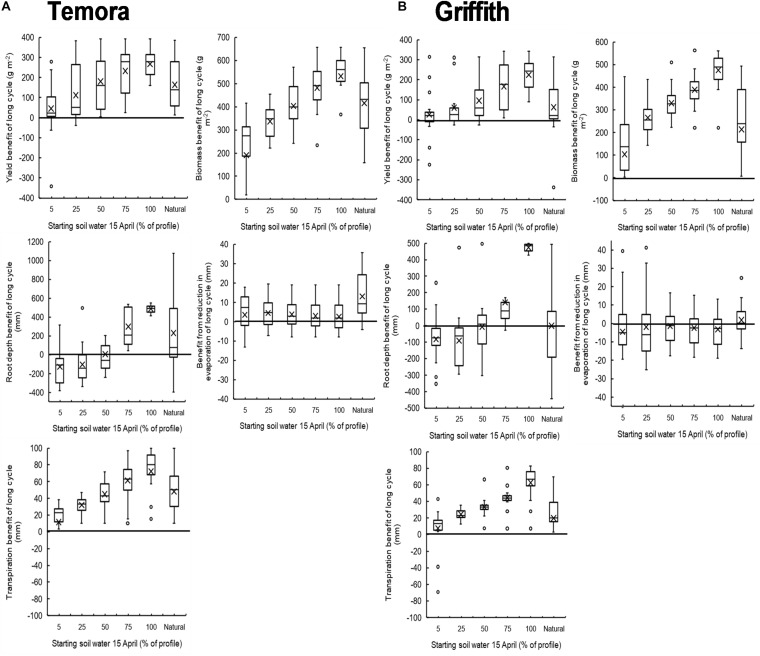
Box and whisker plots showing the simulated yield benefit (1997–2016) of long cycle treatment (Eaglehawk sown 15 April) over the short cycle treatment (Lincoln sown 15 May) at different levels of starting plant available water on 15 April at both Temora **(A)** and Griffith **(B)**.

There was good agreement between simulated and observed yields of long- and short-cycle treatments in 2011 and 2012 (Supplementary Figure S1). When simulated over 19 seasons there was a clear positive relationship between median yield benefit of long-cycle and increasing starting soil water on 15 April at Temora and Griffith (Figure 6). The median yield benefit was ∼100–175 g m^–2^ when the soil profile was 50% filled on 15 April, and ∼250–300 g m^–2^ when the soil profile was 100% filled on 15 April. There was also a positive relationship between biomass, root depth, transpiration benefit and increasing stored soil water on 15 April at both sites. There was no trend for a benefit from a reduction in evaporation in long-cycle treatments in the simulations.

## Discussion

### Environment Determines Yield Response of Long-Cycle Treatments

The yield advantage of long-cycle treatments over short-cycle treatments was strongly related to seasonal pattern of water availability. Requisite environmental conditions for a yield benefit in long-cycle treatments were (a) the presence of accessible deep soil water and (b) low rainfall during the critical period for yield determination in wheat. In 2011 and 2012 there was ∼70 mm more PAW stored at sowing compared to 2015 and 2016. In 2011 and 2012, drought during the critical period for yield determination forced greater reliance on deep soil water for crop growth, and long-cycle treatments were able to access more of this due to longer duration of root growth and thus root depth (Kirkegaard et al., 2015). In seasons where no soil water was available at depth (2015) or enough rain fell during the critical period to meet crop demand from shallow soil layers (2016), long cycle had no yield advantage despite universally lower evaporation, and generally greater dry matter accumulation and higher grain number. Our results support the findings of Peake et al. (2018), who found that long-cycle treatments had a yield advantage only in environments where there was water stress during the critical period. Similarly, Coventry et al. (1993) found a yield advantage in long-cycle treatments in one growing season out of a 2 year experiment. While insufficient nitrogen application may have contributed to the variable yield response in these experiments, above average winter rainfall in 1986 would have filled the soil profile to depth, increasing water availability making greater rooting depth an advantage in long cycle treatments.

The efficiency in converting soil water stored below 1 m into grain yield reported in this study (31 kg ha^–1^mm^–1^) is in close agreement to the marginal water-use efficiency for deep soil water reported by Lilley and Kirkegaard (2007) of 30–36 kg ha^–1^mm^–1^ and Angus and Van Herwaarden (2001) of 33 kg ha^–1^mm^–1^. It is less than the 60 kg/ha. mm reported by Kirkegaard et al. (2007) for efficiency of water use after flowering in one site year where complete terminal stress was established with a rainout shelter, but is close to the average calculated through simulation of 30–40 kg ha^–1^mm^–1^ in the same study. Other authors (e.g., Connor et al., 1992) reported no difference in water extraction at depth between different cycle length, but this may have been due to the presence of sufficient shallow water to meet crop demand as observed here in the 2016 season, or a shallow soil with physical or chemical constraints that limit root depth.

Greater water uptake at depth in 2012 was associated with deeper root growth. Kirkegaard et al. (2015), Figure 3 of that paper report root length density (RLD) for one of the long-cycle treatments (EGA Eaglehawk sown 18 April) and short-cycle treatments (Lincoln sown 17 May) in 2012. There were no roots found in the short-cycle treatment below a depth of 1.4 m, but RLD in the long-cycle treatment exceeded 0.2 cm.cm^–3^ to the deepest sampling depth of 1.8 m. As a result, EGA Eaglehawk was able to extract 21 mm more water than Lincoln. In addition, it was able to convert this water to dry matter more efficiently under terminal drought (3.4 vs. 3.1 g m^–2^ mm^–1^).

Greater rooting depth and deep soil water extraction was achieved in this experiment through a combination of genotype (G, slow development) and management (M, early sowing) resulting in longer life-cycle and thus duration of root growth. However, it only resulted in a yield benefit in specific environments, i.e., in the presence of deep soil water and drought during critical growth period. Other authors have proposed increasing rooting depth by genetic means alone as a useful trait to target for low rainfall environments (Dreccer et al., 2002; Wasson et al., 2012; Rich et al., 2016). However, the magnitude of increase by this mechanism may not be as great as that demonstrated by synergistic G x M interaction here. The findings from the 4-year field experiment are supported by a 19-year simulation experiment conducted at two locations. The simulation study showed that as soil water at the start of the growing season was increased, the yield benefit of long-cycle also increased. As presented in Figure 6, there is considerable variation around the median yield benefit of long-cycle at both locations. This is driven by seasonal variability, and it is likely that in seasons where there was greater long-cycle benefit there was little rainfall in the 30 days preceding the optimal flowering period, and in years where there was little yield advantage over short cycle treatments, there was no spring drought. Our results support other studies that also indicate that deeper roots are only of benefit in environments where soils are able to hold water deep in the profile, rainfall distribution enables deep soil wetting and drought during the critical period and grain filling forces reliance on deep soil water to support growth (Kirkegaard et al., 2007; Lilley and Kirkegaard, 2007, 2016). Our results are limited to a single region with a similar environment. Yield benefits from stored soil water are likely to differ in environments where soil water holding capacities are lower or there is an impediment to root growth, and in environments where rainfall (particularly during the summer fallow) is lower and thus soil water is unlikely to be stored at depth (Lilley and Kirkegaard, 2016).

### Potential for Genetic Improvement of Long-Cycle Cultivars

Our data showed good agreement in the relationship between HI and proportion of water used after flowering proposed by Passioura (1977), who reported that irrespective of the total water supply, HI is optimized when around 30% of the water supply was used after flowering. However, there was no significant difference in the distribution of pre- and post- flowering water use between long- and short-cycle treatments. This implies that imbalance between pre-and post- flowering water use is unlikely to be the reason for low HI in long-cycle treatments observed by other researchers (Gomez-Macpherson and Richards, 1995). Based on our data, it also seems unlikely that competition for assimilates between the elongating stem and developing spike during the critical period reducing grain number is responsible for low HI. Stem weight as a proportion of dry matter was not significantly different, and grain number was never lower in the long-cycle treatments despite equivalent yields. Equivalent yields in 2015 and 2016 were due to lower grain weight, implying greater post-flowering stress in long cycle treatments or an imbalance between source and sink reducing grain weight.

There was an unusual relationship between grain number and yield in the 2 years in which long-cycle treatments showed a yield advantage over short-cycle (Figure 5). Relationships between these two parameters are typically linear (Calderini et al., 1995; Slafer et al., 2005) but both these seasons showed an asymptotic relationship. We hypothesize that this relationship is due to gradual imposition of a sink limitation other than grain number on grain yield as water limited potential yield is approached. It implies an imbalance between source and sink in long-cycle treatments, with sink ultimately limiting yield. Reynolds et al. (2017) demonstrated that substantial yield gains were possible by crossing high dry matter lines (source) with lines selected for strong sink traits (grain number, harvest index, water soluble carbohydrate mobilization) to improve source/sink balance. This raises the possibility that yield could be increased in long-cycle cultivars (which have high biomass through G x M interaction) via genetic improvement of sink traits other than grain number, such as HI, grain size or accumulation of translocatable carbohydrates. Flohr et al. (2018b) demonstrated that historic yield gains in this environment have largely been achieved through increases in HI and that this trait was decoupled from cycle length, supporting the potential for such a breeding strategy to improve yields. HI and grain weight are traits that lend themselves to early generation selection in spaced plants (Fischer and Rebetzke, 2018), and this technique could be used to improve the speed and precision of breeding for high yield in long-cycle cultivars.

### Management for Yield Advantage in Long Cycle Cultivars

Management strategies that improve the capture and efficient use of summer fallow rain (residue retention, summer fallow weed control) are likely to synergize with long-cycle treatments (Kirkegaard et al., 2014). An analysis by Chenu et al. (2013) illustrated that different sowing dates, stored soil water at sowing and genotype development patterns can affect the drought pattern experienced by a crop. It is possible that G × M factors can be further manipulated to increase the yield advantage of long-cycle treatments. Fischer and Armstrong (1990) demonstrated that stored soil water accumulated during fallow periods increases the probability of gaining early sowing opportunities. As practiced in the United States Pacific Northwest (Schillinger, 2016), other authors (Oliver et al., 2010; Flohr et al., 2018a) also surmized that crop rotation and fallow length can be altered in different rainfall zones to increase early sowing and establishment opportunities, and also amount of stored soil water. Farmers intending to use long-cycle cultivars could manage rotations such that soil water accumulation is maximized, i.e., by extending fallow periods to increase both sowing opportunities and the likelihood of achieving a yield advantage with long-cycle. However, this could have negative implications elsewhere in the farming system. In the context of mixed cropping and livestock enterprizes where dual-purpose cropping is possible, long-cycle treatments will provide additional biomass production even from levels of starting water where a grain yield benefit is not observed (Figures 6A,B). Therefore when making decisions regarding when it is profitable to sow long-cycle crops, the value of feed should also be considered rather than for grain harvest alone (Sprague et al., 2018).

Future climates may favor the early sowing x slow developing cultivar x fallow management synergy. Australian agencies (BOM and CSIRO, 2018) reported that while April-October rainfall has declined by 11% since the late 1990s, in the future there will be an increase in intense heavy rainfall events with longer periods spent in drought. Verburg et al. (2012) determined that rainfall in excess of 20–30 mm is needed to infiltrate below the evaporative zone at the soil surface for storage for subsequent crop growth, and an increase in intensity may improve storage. Sowing ultra-long cycle cultivars early into stored soil water could alleviate the on-farm impacts of the decline in April-May rainfall by allowing growers to establish crops using stored soil water from summer fallow rain, rather than relying on the declining in-crop rainfall. In seasons where stored soil water cannot be manipulated with management, or where there is inadequate summer fallow rainfall prior to sowing, growers would be best to sow short-cycle treatments at the optimal sowing date (Flohr et al., 2017).

An analysis of the amount of rainfall required in different environments on soils with different water holding capacities to achieve a yield advantage in long-cycle crops would improve our understanding of environmental suitability. An accurate long-term forecast would enable growers to better decide on long- or short-cycle cultivars (Lilley et al., 2019). Further consideration and analysis is required to determine the legacy effect of growing long-cycle crops that have greater rooting depth and leave the soil in a drier state (Lilley and Kirkegaard, 2016), and on the summer fallow rainfall required to recharge deep stored water.

## Conclusion

Field and simulation results demonstrated that long-cycle cultivars possessed a yield advantage over short-cycle cultivars in seasons where two conditions are met (1) water is stored deep in the soil profile, and (2) where little rain subsequently falls during the critical period for yield determination which increases the reliance on deep water to maintain growth. Early sowing of long-cycle cultivars, and management to maximize accumulation of soil water during fallow periods are complementary practices which will assist to maintain current yield levels in southern Australia under projected future climates. Greater yield gains may be possible in the future by selecting for sink traits other than grain number in long-cycle cultivars.

## Data Availability Statement

The datasets generated for this study are available on request to the corresponding author.

## Author Contributions

JH and BF conceptualized, designed and analysed the experiments and interpreted the results. BF and JH wrote the manuscript and designed and prepared figures and tables. JH and BF managed the collection of data from field experiments, and with assistance from TS, BR, LG, and MB. JH conducted the simulations and BF prepared the figures. JK and JE contributed valuable comment to the final manuscript and interpretation of results.

## Conflict of Interest

The authors declare that the research was conducted in the absence of any commercial or financial relationships that could be construed as a potential conflict of interest.

## Footnotes

1 https://www.apsim.info/Products/APSoil.aspx

## References

[B1] ACAS (2007). *National Variety Trials.* Available online at: http://www.nvtonline.com.au (accessed May 12, 2015).

[B2] Angus J. F. Van Herwaarden A. F. (2001). Increasing water use and water use efficiency in dryland wheat. *Agron. J.* 93 290–298.

[B3] Australian Government (2017). *Climate Data Online [Online].* Available online at: http://www.bom.gov.au/climate/data/ (accessed June 1, 2018).

[B4] Batten G. Khan M. (1987). Effect of time of sowing on grain yield, and nutrient uptake of wheats with contrasting phenology. *Austr. J. Exper. Agric.* 27 881–887.

[B5] Bloomfield M. T. Hunt J. R. Trevaskis B. Ramm K. Hyles J. (2018). Ability of alleles of PPD1 and VRN1 genes to predict flowering time in diverse Australian wheat (*Triticum aestivum*) cultivars in controlled environments. *Crop Past. Sci.* 69 1061–1075.

[B6] BOM and CSIRO (2018). *State of the Climate Report [Online]. © 2018 Commonwealth of Australia.* Available online at: http://www.bom.gov.au/state-of-the-climate/ (accessed May 25, 2019).

[B7] Cai W. J. Cowan T. (2013). Southeast Australia autumn rainfall reduction: a climate-change-induced poleward shift of ocean-atmosphere circulation. *J. Clim.* 26 189–205.

[B8] Calderini D. F. Dreccer M. F. Slafer G. A. (1995). Genetic improvement in wheat yield and associated traits. A re-examination of previous results and the latest trends. *Plant Breed.* 114 108–112.

[B9] Chenu K. Deihimfard R. Chapman S. C. (2013). Large-scale characterization of drought pattern: a continent-wide modelling approach applied to the Australian wheatbelt spatial and temporal trends. *New Phytol.* 198 801–820. 10.1111/nph.12192 23425331

[B10] Connor D. J. Theiveyanathan S. Rimmington G. M. (1992). Development, growth, water-use and yield of a spring and a winter-wheat in response to time of sowing. *Austr. J. Agric. Res.* 43 493–516.

[B11] Coventry D. R. Reeves T. G. Brooke H. D. Cann D. K. (1993). Influence of genotype, sowing date, and seeding rate on wheat development and yield. *Austr. J. Exp. Agric.* 33 751–757.

[B12] Dreccer M. Rodriguez D. Ogbonnaya F. (2002). “Tailoring wheat for marginal environments: a crop modelling study,” in *Proceedings of the 12th Australasian Plant Breeding Conference*, Perth.

[B13] Fischer R. A. (1979). *Growth and Water Limitation To Dryland Wheat Yield In Australia: A Physiological Framework.* Rome: FAO.

[B14] Fischer R. A. (1985). Number of kernels in wheat crops and the influence of solar radiation and temperature. *J. Agric. Sci.* 105 447–461.

[B15] Fischer R. A. (2016). The effect of duration of the vegetative phase in irrigated semi-dwarf spring wheat on phenology, growth and potential yield across sowing dates at low latitude. *Field Crops Res.* 198 188–199.

[B16] Fischer R. A. Armstrong J. S. (1990). Simulation of soil water storage and sowing day probabilities with fallow and no-fallow in southern New South Wales: II. *Stochast. Manag. Tact.* 33 215–240.

[B17] Fischer R. A. Rebetzke G. J. (2018). Indirect selection for potential yield in early-generation, spaced plantings of wheat and other small-grain cereals: a review. *Crop Past. Sci.* 69 439–459.

[B18] Fletcher A. Lawes R. Weeks C. (2016). Crop area increases drive earlier and dry sowing in Western Australia: implications for farming systems. *Crop Past. Sci.* 67 1268–1280.

[B19] Flohr B. M. Hunt J. R. Kirkegaard J. A. Evans J. R. (2017). Water and temperature stress define the optimal flowering period for wheat in south-eastern Australia. *Field Crops Res.* 209 108–119.

[B20] Flohr B. M. Hunt J. R. Kirkegaard J. A. Evans J. R. Lilley J. M. (2018a). Genotype×management strategies to stabilise the flowering time of wheat in the south-eastern Australian wheatbelt. *Crop Past. Sci.* 69 547–560.

[B21] Flohr B. M. Hunt J. R. Kirkegaard J. A. Evans J. R. Swan A. Rheinheimer B. (2018b). Genetic gains in NSW wheat cultivars from 1901 to 2014 as revealed from synchronous flowering during the optimum period. *Eur. J. Agron.* 98 1–13.

[B22] Flohr B. M. Hunt J. R. Kirkegaard J. A. Evans J. R. Trevaskis B. Zwart A. (2018c). Fast winter wheat phenology can stabilise flowering date and maximise grain yield in semi-arid Mediterranean and temperate environments. *Field Crops Res.* 223 12–25.

[B23] Gomez-Macpherson H. Richards R. A. (1995). Effect of sowing time on yield and agronomic characteristics of wheat in south-eastern Australia. *Austr. J. Agric. Res.* 46 1381–1399.

[B24] Goos R. J. Westfall D. G. Ludwick A. E. Goris J. E. (1982). Grain protein-content as an indicator of n-sufficiency for winter-wheat. *Agron. J.* 74 130–133.

[B25] Holford I. C. R. Doyle A. D. Leckie C. C. (1992). Nitrogen response characteristics of wheat-protein in relation to yield responses and their interactions with phosphorus. *Austr. J. Agric. Res.* 43 969–986.

[B26] Holzworth D. P. Huth N. I. Devoil P. G. Zurcher E. J. Herrmann N. I. Mclean G. (2014). APSIM - Evolution towards a new generation of agricultural systems simulation. *Environ. Modell. Softw.* 62 327–350.

[B27] Hunt J. R. (2017). Winter wheat cultivars in Australian farming systems: a review. *Crop Past. Sci.* 68 501–515.

[B28] Hunt J. R. Browne C. Mcbeath T. Verburg K. Craig S. Whitbread A. M. (2013). Summer fallow weed control and residue management impacts on winter crop yield through soil water and N accumulation in a winter-dominant, low rainfall region of southern Australia. *Crop Past. Sci.* 64 922–934.

[B29] Hunt J. R. Kirkegaard J. A. (2011). Re-evaluating the contribution of summer fallow rain to wheat yield in southern Australia. *Crop Past. Sci.* 62 915–929.

[B30] Hunt J. R. Lilley J. M. Trevaskis B. Flohr B. M. Peake A. Fletcher A. (2019). Early sowing systems can boost Australian wheat yields despite recent climate change. *Nat. Clim. Change* 9 244–247.

[B31] Hunt J. R. Swan A. D. Fettell N. A. Breust P. D. Menz I. D. Peoples M. B. (2016). Sheep grazing on crop residues do not reduce crop yields in no-till, controlled traffic farming systems in an equi-seasonal rainfall environment. *Field Crops Res.* 196 22–32.

[B32] Isbell R. F. (2002). *The Australian Soil Classification - Revised Edition.* Collingwood: CSIRO Publishing.

[B33] Jeffrey S. J. Carter J. O. Moodie K. B. Beswick A. R. (2001). Using spatial interpolation to construct a comprehensive archive of Australian climate data. *Environ. Modell. Softw.* 16 309–330.

[B34] Kirkegaard J. A. Gardner P. A. Angus J. F. Koetz E. (1994). Effect of brassica break crops on the growth and yield of wheat. *Austr. J. Agric. Res.* 45 529–545.

[B35] Kirkegaard J. A. Hunt J. R. Mcbeath T. M. Lilley J. M. Moore A. D. Verburg K. (2014). Improving water productivity in the Australian Grains industry - a nationally coordinated approach. *Crop Past. Sci.* 65 583–601.

[B36] Kirkegaard J. A. Lilley J. M. Howe G. N. Graham J. M. (2007). Impact of subsoil water use on wheat yield. *Austr. J. Agric. Res.* 58 303–315.

[B37] Kirkegaard J. A. Lilley J. M. Hunt J. R. Sprague S. J. Ytting N. K. Rasmussen I. S. (2015). Effect of defoliation by grazing or shoot removal on the root growth of field-grown wheat (Triticum aestivum L.). *Crop Past. Sci.* 66 249–259.

[B38] Lilley J. M. Flohr B. M. Whish J. P. M. Farre I. Kirkegaard J. A. (2019). Defining optimal sowing and flowering periods for canola in Australia. *Field Crops Res.* 235 118–128.

[B39] Lilley J. M. Kirkegaard J. A. (2007). Seasonal variation in the value of subsoil water to wheat: simulation studies in southern New South Wales. *Austr. J. Agric. Res.* 58 1115–1128.

[B40] Lilley J. M. Kirkegaard J. A. (2016). Farming system context drives the value of deep wheat roots in semi-arid environments. *J. Exp. Bot.* 67 3665–3681. 10.1093/jxb/erw093 26976814 PMC4896360

[B41] Monteith J. L. (1972). Solar-radiation and productivity in tropical ecosystems. *J. Appl. Ecol.* 9 747–766.

[B42] Oliver Y. M. Robertson M. J. Weeks C. (2010). A new look at an old practice: Benefits from soil water accumulation in long fallows under Mediterranean conditions. *Agric. Water Manag.* 98 291–300.

[B43] Passioura J. B. (1977). Grain-yield, harvest index, and water-use of wheat. *J. Austr. Instit. Agric. Sci.* 43 117–120.

[B44] Peake A. S. Das B. T. Bell K. L. Gardner M. Poole N. (2018). Effect of variable crop duration on grain yield of irrigated spring-wheat when flowering is synchronised. *Field Crops Res.* 228 183–194.

[B45] Penrose L. (1993). Yield of early dryland sowing of wheat with winter and spring habit in southern and central New South Wales. *Austr. J. Exp. Agric.* 33 601–608.

[B46] Penrose L. D. J. Martin R. H. (1997). Comparison of winter habit and photoperiod sensitivity in delaying development in early-sown wheat at a site in New South Wales. *Austr. J. Exp. Agric.* 37 181–190.

[B47] Pook M. Lisson S. Risbey J. Ummenhofer C. C. Mcintosh P. Rebbeck M. (2009). The autumn break for cropping in southeast Australia: trends, synoptic influences and impacts on wheat yield. *Intern. J. Climatol.* 29 2012–2026.

[B48] Pugsley A. T. (1983). The impact of plant physiology on Australian wheat breeding. *Euphytica* 32 743–748.

[B49] Reynolds M. P. Pask A. J. D. Hoppitt W. J. E. Sonder K. Sukumaran S. Molero G. (2017). Strategic crossing of biomass and harvest index—source and sink—achieves genetic gains in wheat. *Euphytica* 213:257 10.1007/s10681-017-2086-y PMC6445510 31187787

[B50] Rich S. M. Wasson A. P. Richards R. A. Katore T. Prashar R. Chowdhary R. (2016). Wheats developed for high yield on stored soil moisture have deep vigorous root systems. *Funct. Plant Biol.* 43 173–188. 10.1071/FP15182 32480451

[B51] Richards R. A. (1991). Crop improvement for temperate Australia: future opportunities. *Field Crops Res.* 26 141–169.

[B52] Riffkin P. A. Evans P. M. Chin J. F. Kearney G. A. (2003). Early-maturing spring wheat outperforms late-maturing winter wheat in the high rainfall environment of south-western Victoria. *Austr. J. Exp. Agric.* 54 193–202.

[B53] Sadras V. Angus J. (2006). Benchmarking water-use efficiency of rainfed wheat in dry environments. *Austr. J. Exp. Agric.* 57 847–856.

[B54] Schillinger W. F. (2016). Seven rainfed wheat rotation systems in a drought-prone Mediterranean climate. *Field Crops Res.* 191 123–130.

[B55] Siddique K. H. M. Tennant D. Perry M. W. Belford R. K. (1990). Water-use and water-use efficiency of old and modern wheat cultivars in a mediterranean-type environment. *Austr. J. Exp. Agric.* 41 431–447.

[B56] Slafer G. A. Araus J. L. Royo C. Del Moral L. F. G. (2005). Promising eco-physiological traits for genetic improvement of cereal yields in Mediterranean environments. *Ann. Appl. Biol.* 146 61–70.

[B57] Sprague S. J. Kirkegaard J. A. Bell L. W. Seymour M. Graham J. Ryan M. (2018). Dual-purpose cereals offer increased productivity across diverse regions of Australia’s high rainfall zone. *Field Crops Res.* 227 119–131.

[B58] Stapper M. Fischer R. A. (1990). Genotype, sowing date and plant spacing influence on high-yielding irrigated wheat in southern New South Wales. II. Growth, yield and nitrogen use. *Austr. J. Exp. Agric.* 41 1021–1041.

[B59] Tottman D. (1987). The decimal code for the growth stages of cereals, with illustrations. *Ann. Appl. Biol.* 110 441–454.

[B60] Verburg K. Bond W. Hunt J. R. (2012). Fallow management in dryland agriculture: Explaining soil water accumulation using a pulse paradigm. *Field Crops Res.* 130 68–79.

[B61] VSN International (2013). *GenStat for Windows*, 18th Edn, Hemel Hempstead: VSN International.

[B62] Wang E. L. Smith C. J. Macdonald B. C. T. Hunt J. R. Xing H. T. Denmead O. T. (2018). Making sense of cosmic-ray soil moisture measurements and eddy covariance data with regard to crop water use and field water balance. *Agric. Water Manag.* 204, 271–280. 10.1016/j.agwat.2018.04.017

[B63] Wasson A. P. Richards R. A. Chatrath R. Misra S. C. Prasad S. V. S. Rebetzke G. J. (2012). Traits and selection strategies to improve root systems and water uptake in water-limited wheat crops. *J. Exp. Bot.* 63 3485–3498. 10.1093/jxb/ers111 22553286

[B64] Zadoks J. C. Chang T. T. Konzak C. F. (1974). A decimal code for the growth stages of cereals. *Weed Res.* 14 415–421.

[B65] Zheng B. Y. Chenu K. Dreccer M. F. Chapman S. C. (2012). Breeding for the future: what are the potential impacts of future frost and heat events on sowing and flowering time requirements for Australian bread wheat (*Triticum aestivium*) varieties? *Glob. Chang. Biol.* 18 2899–2914. 10.1111/j.1365-2486.2012.02724.x 24501066

