# Supplementary material for: Deep Soil Water-Use Determines the Yield Benefit of Long-Cycle Wheat
Source: Front Plant Sci. 2020 May 15;11:548. doi: 10.3389/fpls.2020.00548 (PMC7242739; doi:10.3389/fpls.2020.00548)
Supplement: Supplementary file 1 [file Data_Sheet_1.pdf]

Supplementary Material

Supplementary Table 1. P-values from two-sample t-tests used to determine significant differences between pooled means of long- and short-cycle treatments in Table 5.

	Maturity dry matter (g m^{-2})		Grain yield (g m^{-2})		Harvest Index		Grain number (m^{-2})		Stem weight % of total dry-matter at flowering		Grain weight (mg)	
**Year**	**Long**	**Short**	**Long**	**Short**	**Long**	**Short**	**Long**	**Short**	**Long**	**Short**	**Long**	**Short**
**2011**	0.045		0.020		0.546		< 0.001		0.054		< 0.001	
**2012**	< 0.001		< 0.001		0.775		< 0.001				0.191	
**2015**	0.602		0.330		0.135		0.447		0.992		0.062	
**2016**	0.126		0.139		0.362		< 0.001		0.104		< 0.001	

Supplementary Table 2. Standard deviation around pooled means of long- and short-cycle treatments in Table 5.

	Maturity dry matter (g m^{-2})		Grain yield (g m^{-2})		Harvest Index		Grain number (m^{-2})		Stem weight % of total dry-matter at flowering		Grain weight (mg)	
**Year**	**Long**	**Short**	**Long**	**Short**	**Long**	**Short**	**Long**	**Short**	**Long**	**Short**	**Long**	**Short**
**2011**	83	60	24	34	0.02	0.02	1216	965	5	6	2.4	1.3
**2012**	91	82	39	40	0.03	0.04	1465	850	-	-	2.9	3.0
**2015**	234	246	92	68	0.06	0.06	4953	3148	3	3	5.1	3.8
**2016**	89	218	44	44	0.01	0.08	959	1130	1	2	0.7	2.7

Supplementary Table 3. P-values from two-sample t-tests used to determine significant differences between pooled means of long- and short-cycle treatments in Table 6.

	Crop water use (mm)		Estimated evaporation (mm)		Post-flowering water use (mm)		TE maturity dry matter ($g\ m^{-2}\ mm^{-1}$)		WUE for maturity DM ($g\ m^{-2}\ mm^{-1}$)	
**Year**	**Long**	**Short**	**Long**	**Short**	**Long**	**Short**	**Long**	**Short**	**Long**	**Short**
**2011**	0.258		< 0.001		0.092		0.764		0.156	
**2012**	0.031		< 0.001		0.076		< 0.001		< 0.001	
**2015**	0.005		0.028		0.008		0.730		0.854	
**2016**	0.181		0.142		0.335		0.375		0.276	

Supplementary Table 4. Standard deviation around pooled means of long- and short-cycle treatments in Table 6.

	Crop water use (mm)		Estimated evaporation (mm)		Post-flowering water use (mm)		TE maturity dry matter (g m^{-2} mm^{-1})		WUE for maturity DM (g m^{-2} mm^{-1})	
**Year**	**Long**	**Short**	**Long**	**Short**	**Long**	**Short**	**Long**	**Short**	**Long**	**Short**
**2011**	27	18	10	8	10	16	0.4	0.5	0.3	0.3
**2012**	11	16	3	4	8	13	0.3	0.3	0.2	0.2
**2015**	17	7	9	13	12	22	0.6	0.7	0.5	0.7
**2016**	27	10	3	7	10	7	0.2	0.4	0.1	0.4

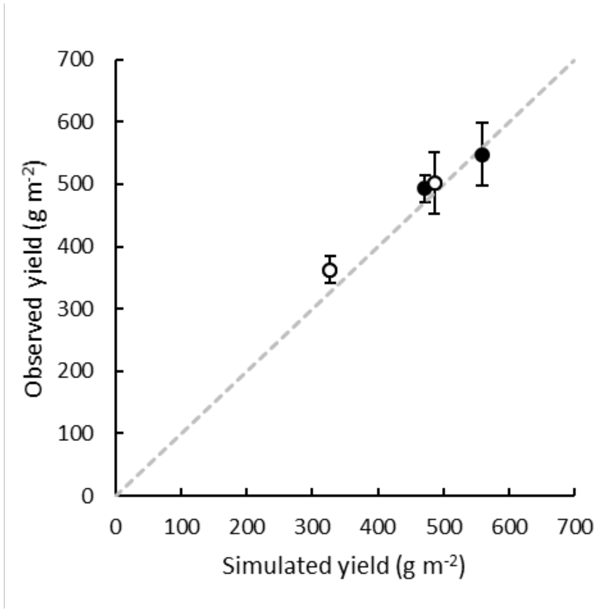

Figure S1. Simulated and observed yields for long (Eaglehawk sown mid-April, ●) and short cycle (Lincoln sown mid-May, ○) treatments at Temora in 2011 and 2012.
